# Multi-parameter simultaneous extraction with a novel microwave sensor based on coupled resonators

**DOI:** 10.1038/s41598-024-72061-3

**Published:** 2024-10-04

**Authors:** Carlos G. Juan, Benjamin Potelon, Anyela Aquino, Héctor García-Martínez, Cédric Quendo

**Affiliations:** 1 Univ. Brest, CNRS, Lab-STICC, UMR CNRS 6285, 29238 Brest, France; 2 Neuroengineering Biomedical Research Group, Institute of Bioengineering, Miguel Hernández University of Elche, 03202 Elche, Spain; 3 Electronic Design and Signal Processing Techniques Research Group, Department of Electronics, Computer Technology and Projects, Technical University of Cartagena, 30202 Cartagena, Spain; 4 IMT Atlantique, Lab-STICC, UMR CNRS 6285, 29238 Brest, France; 5 Facultad de Ingeniería Eléctrica, Electrónica y Telecomunicaciones, Universidad Nacional de Ingeniería, Lima, Perú; 6 Elche Microwave Laboratory, Engineering Research Institute of Elche, Miguel Hernández University of Elche, 03202 Elche, Spain

**Keywords:** Microwaves, Sensor, Permittivity, Multi-parameter measurement, Dataset, Resonators, Electrical and electronic engineering, Electronics, photonics and device physics, Characterization and analytical techniques

## Abstract

This work presents a microwave resonant multi-parameter sensor devoted to the simultaneous extraction of three characteristics of a homogeneous solid sample: its dielectric permittivity, its loss tangent and its thickness. The device is composed of three coupled resonators in two different substrate boards, having the sample between the boards, in a sandwich configuration. Presence of the sample impacts the electrical response of the device, not only influencing resonators, but also by affecting inter-resonator couplings. A method to analyse the response of the device, allowing for the extraction of the desired characteristics of the sample is proposed, as well as an experimental calibration procedure. The model is built upon 990 simulations, calibrated with three reference-samples measurements and then tested over 18 experimental measurements, with good results, thereby validating the multi-parameter sensing approach.

## Introduction

The development of microwave dielectric sensors has raised a constant interest amongst the scientific community during several decades^1^. These sensors, devoted to extract inherent parameters of the Materials Under Test (MUT), such as the complex permittivity (ɛ* = ɛʹ − *j*ɛ″), the position or the orientation, for example, show interesting advantages. Among others, they may provide for contactless measurement, real-time monitoring, low sizes, easy integration with further equipment, cost-effective production, portability, low energy consumption, etc.^2–4^. Due to all these benefits, these sensors are highly sought-after by industries and they find interesting applications in many domains, such as biomedical^5–9^, agricultural^10^, drug testing^11,12^, communications^13^, defect/crack detection^14,15^, or material characterization^16,17^. In the current context, with the potential of the modern additive manufacturing techniques for the electronics industry, the results of which highly depend on the dielectric properties of the materials^18^, the material characterization raises as a considerably interesting application^19,20^.

These characterization methods are traditionally divided into two categories: broadband transmission/reflection methods and resonant sensors. The former show the advantage of broadband characterization, at the expense of a limited sensitivity, especially for low loss materials, such as substrates^21^. The later have more suitable sensitivities for these applications, with the limitation of measurement at discrete frequency points^1^. These circumstances make the resonant methods the usual choice as for solid material characterization for electronics or radio-frequency purposes. These sensors are based on the dependence of their electrical response on the dielectric properties and geometry of the MUT when placed in their vicinity^22^. The changes in the properties of the MUT can be seen through several indicators of the measured scattering parameters of the response of the device, such as resonance frequency^23^, insertion/return loss^24^, quality factor^19,22^ or phase^25^.

After the achievement of unprecedented high sensitivities^26–32^, particularly through coupled structures^33^, one of the main challenges nowadays is the selectivity or the possibility to discern different parameters or characteristics from the MUT^34,35^. As an example, thickness characterization of solid slabs is essential for wafer manufacturing and electronic circuitry applications^36^. As widely known, the final performance of the manufactured device will strongly depend also on the relative dielectric permittivity (ɛ_r_) and dielectric losses (loss tangent, tan δ = ɛ″/ɛʹ) of the material. Consequently, the possibility of simultaneous extraction of these three features from an unknown or experimental slab appears as starkly desirable. Many works have been recently done pursuing this point. The simultaneous extraction of two features with resonant sensors has already been demonstrated for solid samples^3,37,38^ and for liquid samples^39–41^.

Focusing on solid materials, recent works showed effective determination of two parameters by several techniques. In a prior work, the use of a single resonator in a sandwich configuration, coupled to the input/output lines through the solid sample, was proposed. It was shown that the dielectric permittivity and the loss tangent can be separately determined by means of the variations of the resonant frequency and the unloaded quality factor, respectively^3^. Nogueira et al. proposed a complementary split-ring resonator (CSRR) sensor for the characterization of dielectric slabs with a similar parameter extraction strategy^42^. Alimenti et al. particularized this dielectric permittivity and loss tangent extraction technique to 3-D-printing materials with a split-ring resonator (SRR) sensor^37^. Considering the determination of the dielectric permittivity and the thickness of the sample, it was shown that their cross-influence in the measurement process hinders their separate extraction^4,43^. As a consequence, these parameters need to be simultaneously extracted by analytical methods. Under this approach, Sun et al. proposed a sensor made of two coupled SRR for simultaneously measuring the dielectric permittivity and thickness of dielectric slabs by substituting the frequencies of the two observed poles into analytical expressions obtained by curve fitting^44^. In a more recent work, Wu and Zhao showed a sensor composed of two magnetic-*LC* resonators etched at each branch of a splitter/combiner, able of simultaneously determine the dielectric permittivity and thickness of solid samples by introducing the measured odd- and even-mode resonant frequencies into experimental analytical expressions^38^. However, despite these progresses as for dual parameter extraction, the simultaneous extraction of three features from the sample with this kind of sensors has not been fully achieved yet, not even applying sophisticated artificial intelligence techniques^45^.

**Fig. 1:**
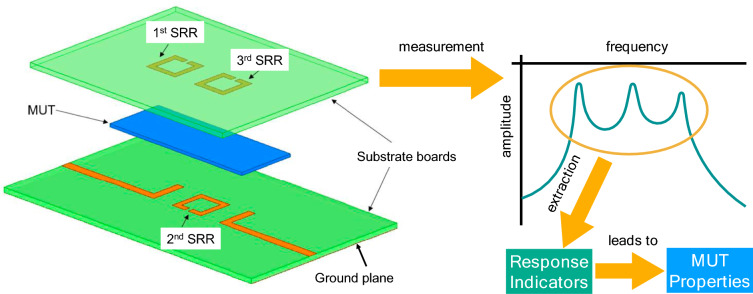
General concept of the proposed multi-parameter sensor.

These last advancements show that parameters having an almost-independent effect on the measurement, such as the dielectric permittivity and loss tangent, can be extracted by separately analysing the proper features of the response of sensors composed of a single resonant element (e.g. Ref.^3^). For extracting two parameters with a cross-influence on the measurement, the currently available research works required at least two resonant elements, in addition to analytical expressions for simultaneous (and not separate) extraction (e.g. Ref.^44^). From these observations, and considering the above-mentioned interest on the simultaneous extraction of the trio of parameters (dielectric permittivity, loss tangent, thickness), in this work we hold the hypothesis that these parameters can be all simultaneously determined with a sensor involving three coupled resonators and a proper analytical extraction method. This hypothesis is based on the idea that, when the presence of the sample is impacting the response of the resonators and their couplings, if an enough amount of information is available from three resonant peaks, we might be able to extract three different features from the sample. Proving such a hypothesis would pave the way for the general analytical multi-parameter extraction with sensors involving multiple coupled resonators. To test it, a simple implementation of the three-resonator sensor in a sandwich configuration (due to its already proven convenience^3^) and an analytical data extraction method based on multi-stage curve fitting from a large set of simulated data is proposed. The general idea is illustrated in Fig. 1.

The rest of the article is organized as it follows. The section “Design procedures” will show the physical design of the sensor followed by an analysis of its electromagnetic response, with special emphasis on the impact of the properties of the sample. From this response, several indicators will be defined, to be related to the properties of the sample through a system of equations composing a direct problem. Then, the inverse solver of this direct problem will allow for the extraction of the unknown properties of the sample from the indicators in the response of the sensor. The section “Results” will discuss the fabrication of the sensor and its experimental assessment, showing the results of the experimental measurement of 18 samples for verification. The section “Discussion” will evaluate the results, outlining the main ideas and discussing the limitations of this work. Finally, the section “Methods” will provide a detailed explanation of the experimental procedure for the calibration of the sensor and the measurements.

## Design procedures

### Sensor design

The sensor under study involves three mutually-coupled Split-Ring Resonators (SRR, also known as Open-Loop Resonators, OLR), in addition to two input/output (I/O) lines, being all these elements etched at two different layers. The structure is composed of two substrate boards, one upon the other one. The top substrate board has no copper coat on its top face, whilst the 1st and 3rd SRR are etched on its copper-clad bottom face. The I/O lines and the 2nd SRR are etched on the top face of the bottom substrate board, serving the copper-clad bottom face of this substrate board as ground plane. With this configuration, the sample or Material Under Test (MUT) is sandwiched between the two substrate boards, housed between the layer containing the I/O lines and the 2nd SRR and the layer containing the 1st and 3rd SRR. Figure 1 (left) depicts an exploded view of the sensing structure.

**Fig. 2:**
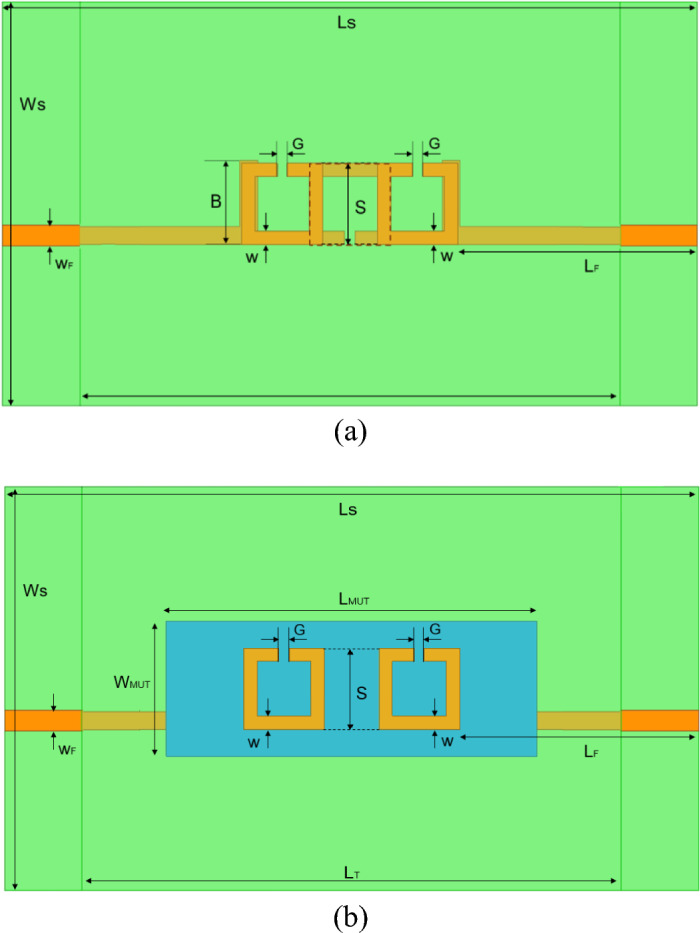
Design of the proposed sensor. (**a**) Top view including dimensions, without MUT. (**b**) Top view including dimensions, with MUT.

The different etched elements are placed so that the two parallel non-split lines of the 1st and the 3rd SRR are parallel, on a vertical projection, to each one of the left and right parallel non-split lines of the 2nd SRR, respectively, as well as to the final 90°-rotated sections of the left and right I/O lines, respectively, as shown in Fig. 2a. This way, when travelling from the input to the output of the structure, the electromagnetic waves must travel across multiple couplings, viz. input line–1st SRR, 1st SRR–2nd SRR, 1st SRR–3rd SRR, 2nd SRR–3rd SRR, 3rd SRR–output line. This configuration makes all the consecutive elements in the structure mutually coupled, in addition to other likely cross-couplings. Having the MUT sandwiched within this vertical space, the fringing fields in the proximity couplings are affected by the MUT during each one of these hops between top and bottom layers, being all these couplings thereby affected by the dielectric properties and dimensions (particularly thickness) of the MUT. The fact that the presence of the MUT has an impact on global effects, such as cross-couplings, and local effects, such as single couplings, determining all these effects the final response of the sensor, should be beneficial for separating the effects of the different characteristics of the MUT (dielectric properties and thickness) and identifying them all individually. To put it in a nutshell, this structure seeks to maximize the influence of the properties and dimensions of the MUT in the propagation of the waves by forcing the waves to travel across the MUT multiple times. This effect is expected to leave an imprint in the transmitted scattering parameter (from the input to the output of the device) notable enough to allow for extraction of multiple parameters or properties of each MUT.

In order to test this idea, an implementation of the sensor is proposed. Three SRR were selected with the same dimensions (and hence the same resonance frequency) with the aim of simplifying the analysis. As operating frequency for each individual resonator, 2 GHz was selected. This choice results in a comfortable frequency for performing radio-frequency (RF) measurements with conventional equipment and it yields wieldy values of all the dimensions in the structure so that a proper, accurate fabrication with micro-drilling machine can be ensured. The impedance of the I/O lines was set to 50 Ω to provide for matched measurements with a Vector Network Analyzer (VNA). The substrate chosen was 1600 μm thick FR4 (ɛ_r_ = 4.4, tan δ = 0.02), one of the most commonly available RF substrates, with ∼ 35 μm thick copper coat. The top FR4 board was slightly shorter than the bottom FR4 board so that subminiature version A (SMA) connectors could be properly housed and soldered, thus providing for VNA-driven measurements. All these decisions were taken with the purpose of achieving a generic implementation of the sensing structure that allows us to test our research hypothesis. The optimization of all these aspects is left as a future challenge.

It should be remarked that a sensor involving multiple coupled resonators was chosen under the assumption that coupled structures make the extraction of three features from the MUT easier. In this sense, it is well known that the final response of microwave filters is considerably sensitive to the couplings between the resonant elements^46^. The proposed structure intends to leverage this effect for sensing purposes by analysing the potential changes in the different poles of the response due to variations in the properties of the MUT. However, the plane response characteristic of matched filters could potentially hamper the identification and processing of these poles. Consequently, the sensor was designed as an unmatched filter, the response of which shows three easily distinguishable poles, thereby facilitating the processing and analytical extraction of the final data.

Considering all these parameters and decisions, the resulting dimensions of the final design of the sensor, depicted in Fig. 2a (without MUT) and Fig. 2b (with MUT), are summarized in Table 1. It is worthwhile to notice that, considering the resulting dimensions of the SRR, minimum dimensions of the MUT of 55 × 20 mm^2^ must be ensured for proper measurement homogeneously covering all the sensing space. In this sense, although the thickness (or height) is one of the a priori unknown parameters to be extracted, a certain secure range must also be ensured to avoid problems with the couplings. To remain cautious, we selected the reasonable 0.8–1.2 mm range, although a thorough study of this range and its possibilities raises as an interesting future research point. As a visual reference, the MUT appearing in Figs. 1 (left) and 2b has the minimum dimensions as for *L*_*MUT*_ and *W*_*MUT*_, and a value of *H*_*MUT*_ = 1.0 mm, falling in the middle of the height range.

**Table 1:** Summary of design dimensions for the proposed sensing structure.

Dimension	Value (mm)	Description
*L*_*S*_	135.0	Bottom substrate length
*L*_*T*_	95.0	Top substrate length
*W*_*S*_	80.0	Top and bottom substrates width
*L*_*F*_	56.61	Feed I/O line length
*W*_*F*_	3.1	Feed I/O line width
*B*	12.1	Coupling stub length
*G*	1.5	SRR open-end gap
*S*	12.0	SRR side length
*W*	2.0	SRR line width
*h*	1.6	Substrate thickness
*th*	∼ 0.035	Copper coat thickness
*L*_*MUT*_	> 55.0	MUT minimum length
*W*_*MUT*_	> 20.0	MUT minimum width
*H*_*MUT*_	0.8 < *H*_*MUT*_ < 1.2	MUT acceptable height range

When designing each SRR, a squared OLR was selected due to its previously proven convenience^3,22^. The SRR were tuned to show the first resonance at 2 GHz when individually etched on an isolated FR4 board without MUT (only air) upon it, resulting in the dimensions shown in Table 1. Essentially, these open-loop resonators behave as a parallel *RLC* circuit consisting of an inductance induced by the circulating current in the loop and an effective capacitance resulting from the contribution of the distributed capacitance through the line combined with the capacitive gap breaking the loop. This effective capacitance shows a firm dependence on the effective complex permittivity of the surrounding media^47^, which depends on the substrate (fixed) and the MUT (variable) with the proposed configuration. A change in the dielectric properties in the MUT should be seen as a change in the effective self-capacitance of the resonator. If no significant changes are observed in the inductance (which is acceptable for dielectric, non-magnetic samples, such as the ones considered in this study), these variations in the MUT properties should yield changes in the resonant behaviour of the SRR. This, in addition to the simultaneous effect likely to appear in all the direct and even cross-couplings of the structure (being their capacitive part affected by the changes of the dielectric properties of the MUT), supports the idea that the changes in the properties of the MUT will leave a remarkable imprint in the transmission parameter of the structure, noticeable enough to extract its main properties.

Specifically, this sensor will be tested for the determination of the values of the *H*_*MUT*_, ɛ_r_ and tan δ of the sample. Particularly, in a summarized way the effects of the variations in *H*_*MUT*_ are expected to have an impact on the resonance frequencies of the resonators and the couplings, thereby affecting the different bandwidths in the response of the device (which are also involved in the computation of the unloaded quality factors); the effects of the variations in ɛ_r_ are expected to have also an impact on the resonance frequencies and bandwidths; and the effects of the variations in tan δ are expected to have an impact in the general losses of the response, i.e. in the amplitude levels (also involved in the computation of the unloaded quality factors). It is easy to see that the influence of the properties of the sample (*H*_*MUT*_, ɛ_r_ and tan δ) is distributed through the response of the sensor, affecting each one of these MUT properties to several metrics or indicators that could be derived from the response of the sensor. However, these influences are overlapped between them and between the affected indicators, which hampers the straight determination of the sample properties from the possible raw indicators computed in the sensor response. This means that a careful analysis of the electromagnetic response of the sensor is required in order to identify the suitable indicators, and a sample properties extraction strategy from the proposed indicators needs to be defined as well.

### Electromagnetic response analysis and indicators definition

**Fig. 3:**
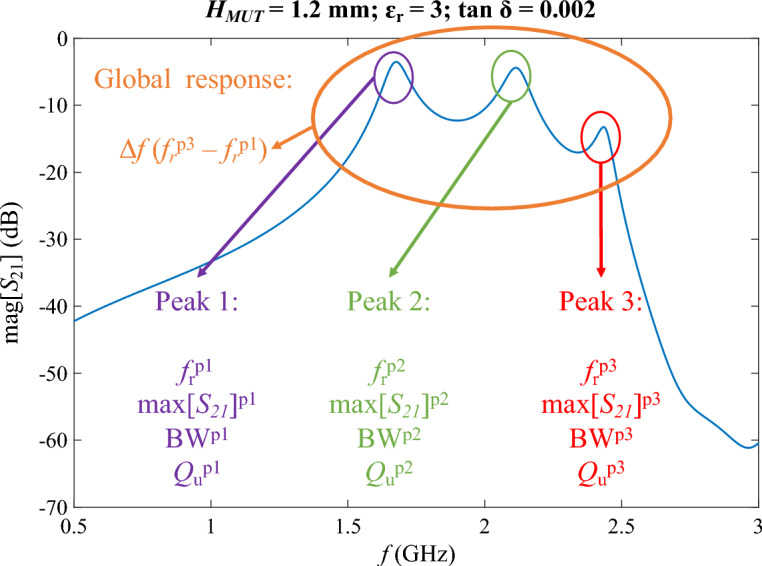
Simulated response of the proposed sensor with a MUT with *H*_*MUT*_ = 1.2 mm, ɛ_r_ = 3 and tan δ = 0.002, and the different possible indicators to be tracked.

The proposed sensor is expected to show an electrical response in terms of the scattering parameters characterized by three peaks in the transmission parameter *S*_21_, given its non-matched filter condition. The exact characteristics of this response will depend on the characteristics of the MUT or sample that was sandwiched during the measurement. In order to define a proper measurement strategy, this response was extensively studied by simulations. As an example, Fig. 3 shows the response obtained by simulation when a MUT with *H*_*MUT*_ = 1.2 mm, ɛ_r_ = 3 and tan δ = 0.002 was involved. The three peaks are clearly visible. The measurement principle is based on the identification of certain indicators from this response that can be used to extract the properties of the MUT (*H*_*MUT*_, ɛ_r_ and tan δ in the proposed case). Analysing the response in Fig. 3, multiple candidate indicators can be spotted. On the one hand, several indicators from each individual peak p*i* (where *i* = 1, 2, 3) can be analysed, such as the associated resonance frequency (*f*_r_^p*i*^), the associated maximum (max[*S*_21_]^p*i*^), the associated bandwidth (*BW*^p*i*^, computed at − 3 dB fall from maximum or any other convenient fraction) or the associated unloaded quality factor (*Q*_u_^p*i*^). On the other hand, certain indicators for the global response can also be considered, such as for example the global bandwidth ∆*f* defined as the frequency range between the first and the last resonance frequency (∆*f* = *f*_r_^p3^ − *f*_r_^p1^). Other global indicators, not considered here for the sake of reducing the dimensions of the problem, could be used, like the lowest level of the *S*_21_ within the bandwidth, other combinations of frequencies, etc.

From all these candidate indicators, which can be directly computed from the response of the sensor, the purpose is to find a suitable group from which the characteristics of the MUT (*H*_*MUT*_, ɛ_r_ and tan δ, supposed to be unknown in a measurement context) can be derived. Aiming at the extraction of three unknown characteristics of the MUT, only three indicators from the response of the sensor must be selected in order to avoid overfitting the problem (it should be noted that if more than three indicators were chosen an overdetermined equation system would appear, potentially leading to null solutions). This may be a crucial step since indicator choice is important both from the individual characteristic determination point of view and also collectively in the indicators–characteristics relationships set. Indeed, sensitivity of each indicator to each characteristic of the MUT should be sought but also enough complementarity of the indicators should be garnered in order to extract all the characteristics from their distributed influences over all the indicators.

In this first exploratory study, not aiming at an optimization of the system but at testing the multi-measurement hypothesis, the selection of these indicators was based on the current understanding of dielectric sensors and their relationships according to information theory. In this sense, indicators from different resonance peaks (or global indicators) should be selected, being the three indicators of different kinds, trying to gather as much information as possible. Also, the indicators should be representative of the three sample properties to be extracted. Still under this philosophy, the resonance frequencies are expected to be mostly affected by the relative permittivity of the sample, the losses in the sample are expected to have an impact on the quality factors, and the local or global bandwidths are expected to be impacted by the couplings between the resonators, which indeed depend on the separation distance, i.e. *H*_*MUT*_, among other likely but not exclusive relationships. This discussion led to the selection of *set_indicators* = (*Q*_u_^p1^, *f*_r_^p3^, ∆*f*) as the set of sensor response indicators from those in Fig. 3, which will be used to extract the unknown characteristics of the sample in this particular implementation. The challenge now lies upon the finding of suitable relationships between the indicators and the sample properties, which will be discussed in the following subsections.

### Direct problem

For this first approach, the initial purpose is the definition of three functions relating each one of the three selected indicators with the three unknown characteristics of the MUT. Formally, with a generic approach, considering the three selected indicators as *Indicator1*, *Indicator2* and *Indicator3*, and the three functions as *X*, *Y* and *Z*, the objective is the definition of the following relationships:

1.a $$Indicator1=X\left({H}_{MUT}, {\varepsilon }_{\text{r}}, \text{tan}\delta \right),$$

1.b $$Indicator2=Y\left({H}_{MUT}, {\varepsilon }_{\text{r}}, \text{tan}\delta \right),$$

1.c $$Indicator3=Z\left({H}_{MUT}, {\varepsilon }_{\text{r}}, \text{tan}\delta \right),$$

The set of Eqs. (1.a–1.c) composes the direct problem of the proposed measurement procedure. Once this direct problem has been accurately defined (*Indicator1*, *Indicator2*, *Indicator3*, *X*, *Y* and *Z* are well defined), the unknown characteristics of the MUT can be obtained by computing and solving the inverse relationships in Eqs. (1.a–1.c), i.e., solving the inverse problem, allowing for unknown sample characterization.

The definition and solution of the direct problem requires, on the one hand, the selection of the three indicators to be involved in Eqs. (1.a–1.c) from the ones shown in Fig. 3 and, on the other hand, the definition of the direct problem functions *X*, *Y* and *Z* in Eqs. (1.a–1.c). Having selected the indicators for our case as *set_indicators* = (*Indicator1* = *Q*_u_^p1^, *Indicator2* = *f*_r_^p3^, *Indicator3* = ∆*f*), as shown in the last subsection, let us now approach the definition of the functions. In this exploratory study, without any warranty of optimization but with testing purposes, building these functions with a three-stage quadratic curve fitting is proposed, as shown next. Polynomial functions are selected due to their ease of operation, whilst a quadratic approach is considered as a trade-off between the lack of accuracy of the linear fit and the exponential increase in complexity of higher orders.

For trustable curve fitting, a remarkable number of points needs to be garnered. With this purpose, a comprehensive set of full-wave simulations of the proposed sensor with ANSYS HFSS 2019 R2 software including a large set of simulated solid samples was run. The envisioned application of this multi-parameter sensor is the full characterization of solid materials for RF manufacturing context. Therefore, in our simulations refined ranges for the properties of the sample were considered, approaching the final measurement scenario, thereby seeking for valid functions to solve experimental measurements. Thus, the ɛ_r_ ranged from 1 to 10 in steps of 1, the tan δ from 0 to 0.01 in steps of 0.001 and the *H*_*MUT*_ from 0.8 to 1.2 mm in steps of 0.05 mm, yielding a total amount of 990 simulations. The raw data (*.s2p files) of all these simulations, which could be useful for future studies, are freely available in the repository^48^.

**Fig. 4:**
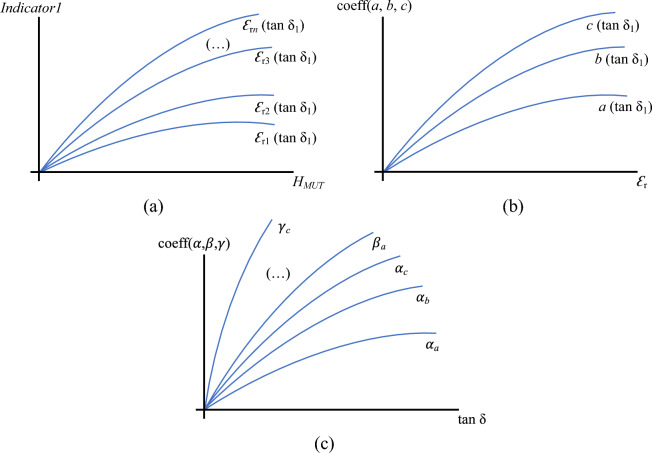
Illustration of the three-stage curve fitting (Latin-letter coefficients are the resulting fitting coefficients from the first stage against *H*_*MUT*_, fitted against ɛ_r_ in the second stage, always with a fixed value of tan δ; Greek-letter coefficients are the resulting fitting coefficients from the second stage against ɛ_r_, fitted against tan δ in the third stage). (**a**) First stage. (**b**) Second stage. (**c**) Third stage.

After running the simulations, all the indicators of each simulated response (all the indicators shown Fig. 3) were computed and saved in a database, which was later used to fit the direct problem curves (file “all_indicators.txt” in the data available in the repository^48^). The fitting is independent for each indicator of the response of the sensor. For example, considering *Indicator1* in Eqs. (1.a–1.c), the proposed first fitting stage consists in taking a fixed value for tan δ and selecting all the rows from the database with this value of tan δ. Let tan δ_1_ be this fixed value. The result is a set of *Indicator1* values, each one associated to a (*H*_*MUT*_, ɛ_r_, tan δ_1_) point, being *H*_*MUT*_ and ɛ_r_ variable. This allows to define different *Indicator1*–*H*_*MUT*_ curves for the different values of ɛ_r_ (ɛ_r1_, ɛ_r2_, …, ɛ_r*n*_), being all of them associated to the value tan δ_1_. This is illustrated in Fig. 4a. Each one of these curves can be fitted to a quadratic polynomial by least squares regression, obtaining the fitting coefficients *a*_*i*_, *b*_*i*_ and *c*_i_, as shown in:

2 $$\begin{array}{c}Indicator1\left({\varepsilon }_{\text{r}1},{\text{tan}\delta }_{1}\right) ={a}_{1}{{H}_{MUT}}^{2}+{b}_{1}{H}_{MUT}+{c}_{1}\\ Indicator1\left({\varepsilon }_{\text{r}2},{\text{tan}\delta }_{1}\right) ={a}_{2}{{H}_{MUT}}^{2}+{b}_{2}{H}_{MUT}+{c}_{2}\\ \begin{array}{c}\vdots \\ Indicator1\left({\varepsilon }_{\text{r}n},{\text{tan}\delta }_{1}\right) ={a}_{n}{{H}_{MUT}}^{2}+{b}_{n}{H}_{MUT}+{c}_{n}\end{array}\end{array}.$$

In these curves, the coefficients (*a*_1_, *b*_1_, *c*_1_) are associated to the fixed values of ɛ_r1_ and tan δ_1_, (*a*_2_, *b*_2_, *c*_2_) are associated to the fixed values of ɛ_r2_ and tan δ_1_, and so on, and all of them describe the variations of *Indicator1* according to *H*_*MUT*_ for the corresponding fixed values of ɛ_r_ and tan δ. After all this process, a set of coefficients *a*_*i*_, *b*_*i*_ and *c*_i_ are obtained, all of them associated to the value of tan δ_1_ and to different values of ɛ_r_. In the second fitting stage, all these coefficients (sorted as *a*-, *b-* and *c*-type) are plotted against their corresponding ɛ_r_ values, and the three resulting curves (one per coefficient type) are fitted to new quadratic polynomials as defined in (this process is illustrated in Fig. 4b):

3 $$\begin{array}{c}a\left({\text{tan}\delta }_{1}\right) ={\alpha }_{a}{{\varepsilon }_{\text{r}}}^{2}+{\beta }_{a}{\varepsilon }_{\text{r}}+{\gamma }_{a}\\ b\left({\text{tan}\delta }_{1}\right) ={\alpha }_{b}{{\varepsilon }_{\text{r}}}^{2}+{\beta }_{b}{\varepsilon }_{\text{r}}+{\gamma }_{b}\\ c\left({\text{tan}\delta }_{1}\right) ={\alpha }_{c}{{\varepsilon }_{\text{r}}}^{2}+{\beta }_{c}{\varepsilon }_{\text{r}}+{\gamma }_{c}\end{array}.$$

At this point, considering the fixed value tan δ_1_, the direct problem for *Indicator1* involving the variations due to changes in the *H*_*MUT*_ and ɛ_r_ of the sample can be expressed by substituting Eq. (3) into Eq. (2) as:

4 $$Indicator1\left({\text{tan}\delta }_{1}\right)=\left({\alpha }_{a}{{\varepsilon }_{\text{r}}}^{2}+{\beta }_{a}{\varepsilon }_{\text{r}}+{\gamma }_{a}\right){{H}_{MUT}}^{2}+\left({\alpha }_{b}{{\varepsilon }_{\text{r}}}^{2}+{\beta }_{b}{\varepsilon }_{\text{r}}+{\gamma }_{b}\right){H}_{MUT}+{\alpha }_{c}{{\varepsilon }_{\text{r}}}^{2}+{\beta }_{c}{\varepsilon }_{\text{r}}+{\gamma }_{c}.$$

Then, these two fitting stages are repeated for all the values of tan δ so that a set of stage-2 coefficients are obtained depending on the value of tan δ, sorted by α_*a*_-, α_*b*_-, α_*c*_-, β_*a*_-, β_*b*_-, β_*c*_-, γ_*a*_-, γ_*b*_- and γ_*c*_-type. With a similar procedure, these coefficients are plotted against the tan δ and the resulting curves are fitted during the third fitting stage (illustrated in Fig. 4c), yielding:

5 $$\begin{array}{c}{\alpha }_{a} ={A}_{{\alpha }_{a}}{\text{tan}\delta }^{2}+{B}_{{\alpha }_{a}}\text{tan}\delta +{C}_{{\alpha }_{a}}\\ {\alpha }_{b} ={A}_{{\alpha }_{b}}{\text{tan}\delta }^{2}+{B}_{{\alpha }_{b}}\text{tan}\delta +{C}_{{\alpha }_{b}}\\ \begin{array}{c}\begin{array}{c}{\alpha }_{c} ={A}_{{\alpha }_{c}}{\text{tan}\delta }^{2}+{B}_{{\alpha }_{c}}\text{tan}\delta +{C}_{{\alpha }_{c}}\\ \begin{array}{c}{\beta }_{a} ={A}_{{\beta }_{a}}{\text{tan}\delta }^{2}+{B}_{{\beta }_{a}}\text{tan}\delta +{C}_{{\beta }_{a}}\\ \vdots \end{array}\end{array}\\ {\gamma }_{c} ={A}_{{\gamma }_{c}}{\text{tan}\delta }^{2}+{B}_{{\gamma }_{c}}\text{tan}\delta +{C}_{{\gamma }_{c}}\end{array}\end{array}.$$

Finally, substituting Eq. (5) into Eq. (4) leads to the definition of the direct problem for *Indicator1*:

6 $$Indicator1=\left[\left({A}_{{\alpha }_{a}}{\text{tan}\delta }^{2}+{B}_{{\alpha }_{a}}\text{tan}\delta +{C}_{{\alpha }_{a}}\right){{\varepsilon }_{\text{r}}}^{2}+\left({A}_{{\beta }_{a}}{\text{tan}\delta }^{2}+{B}_{{\beta }_{a}}\text{tan}\delta +{C}_{{\beta }_{a}}\right){\varepsilon }_{\text{r}}+{A}_{{\gamma }_{a}}{\text{tan}\delta }^{2}+{B}_{{\gamma }_{a}}\text{tan}\delta +{C}_{{\gamma }_{a}}\right]{{H}_{MUT}}^{2}+\left[\left({A}_{{\alpha }_{b}}{\text{tan}\delta }^{2}+{B}_{{\alpha }_{b}}\text{tan}\delta +{C}_{{\alpha }_{b}}\right){{\varepsilon }_{\text{r}}}^{2}+\left({A}_{{\beta }_{b}}{\text{tan}\delta }^{2}+{B}_{{\beta }_{b}}\text{tan}\delta +{C}_{{\beta }_{b}}\right){\varepsilon }_{\text{r}}+{A}_{{\gamma }_{b}}{\text{tan}\delta }^{2}+{B}_{{\gamma }_{b}}\text{tan}\delta +{C}_{{\gamma }_{b}}\right]{H}_{MUT}+ \left({A}_{{\alpha }_{c}}{\text{tan}\delta }^{2}+{B}_{{\alpha }_{c}}\text{tan}\delta +{C}_{{\alpha }_{c}}\right){{\varepsilon }_{\text{r}}}^{2}+\left({A}_{{\beta }_{c}}{\text{tan}\delta }^{2}+{B}_{{\beta }_{c}}\text{tan}\delta +{C}_{{\beta }_{c}}\right){\varepsilon }_{\text{r}}+{A}_{{\gamma }_{c}}{\text{tan}\delta }^{2}+{B}_{{\gamma }_{c}}\text{tan}\delta +{C}_{{\gamma }_{c}}.$$

This procedure implies obtaining a total amount of 27 fitting coefficients, as shown in Eq. (6), for each one of the indicators in the definition of the direct problem shown in Eqs. (1.a–1.c). The fitting coefficients for the 13 possible indicators in Fig. 3 were computed and saved in a new database with 13 rows and 27 columns (file “coefficients_for_all_indicators.txt” in the data available in the repository^48^). This will allow to easily explore different combinations and ways of solving the direct problem in Eqs. (1.a–1.c). It should also be noticed that this procedure is only possible with discrete values that can be grouped for the properties of the MUT, which is difficult to achieve with experimental measurements (considering an amount of measurements large enough for trustable curve fitting), which forces the process to be made by simulations.

**Fig. 5:**
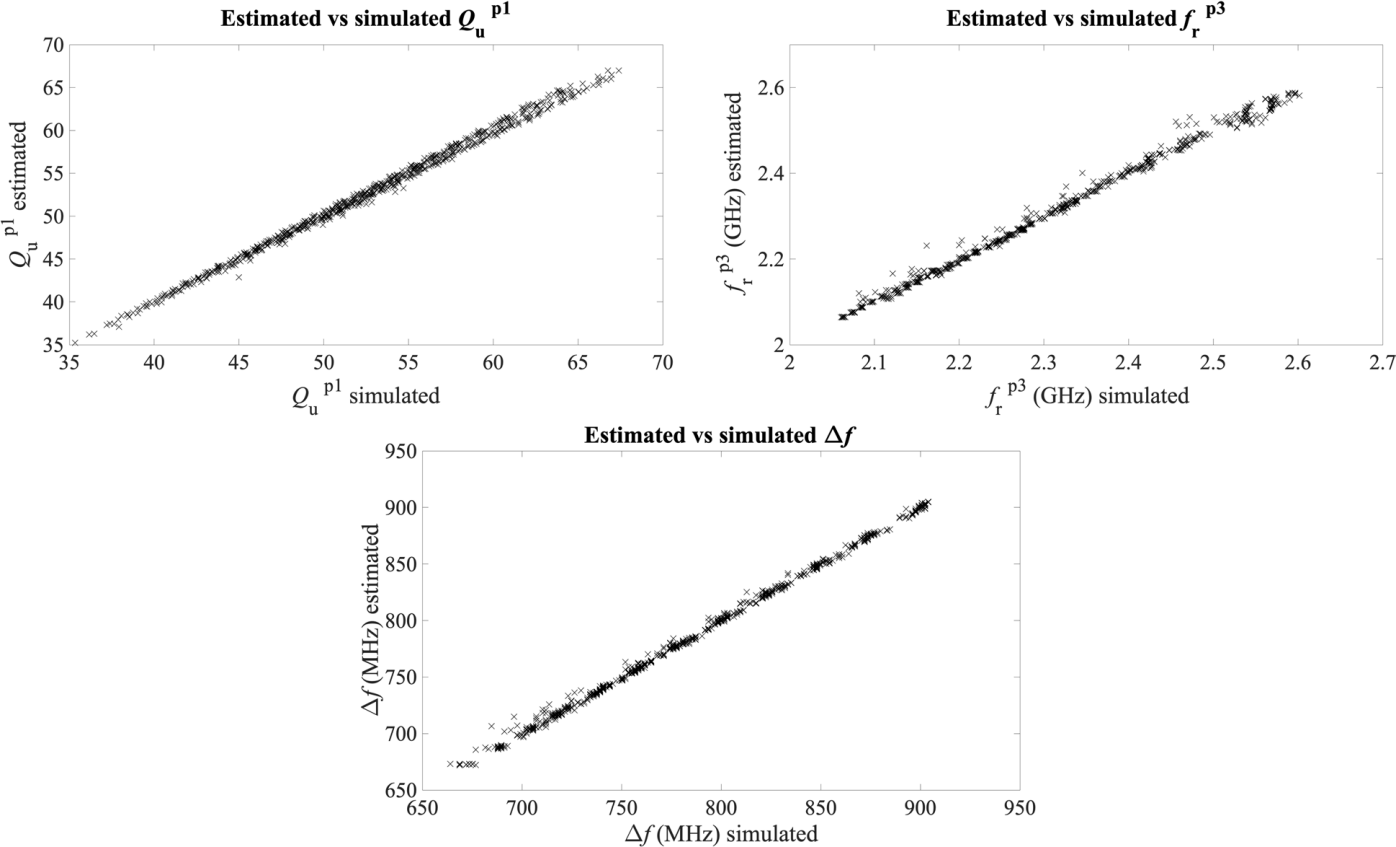
Results for the solution of the direct problem in simulations with *set_indicators*.

After the fitting procedure, with the indicators in *set_indicators* for our case and the corresponding fitting coefficients, the direct problem in Eqs. (1.a–1.c) could be built and solved for all the simulations. The solution implies assuming the properties of the sample (*H*_*MUT*_, ɛ_r_, tan δ) as known values, and estimating the associated values of (*Indicator1*, *Indicator2*, *Indicator3*) = (*Q*_u_^p1^, *f*_r_^p3^, ∆*f*) by solving Eqs. (1.a–1.c). Then, the results can be checked by seeing the actual result of each simulation. The results of the solution of the direct problem for the indicators in *set_indicators* are shown in Fig. 5. The obtained coefficients of determination for *Q*_u_^p1^, *f*_r_^p3^ and ∆*f* are 0.9962, 0.9947 and 0.9979, respectively.

### Inverse problem

Having defined and solved the direct problem, which allows to predict the expected indicators of the response of the sensor provided that the properties of the sample are known, the capabilities of the system for solving the inverse problem were tested. This inverse problem should allow us to extract the unknown properties of the sample provided that the corresponding measurement has been made and the indicators of the response of the sensor have already been computed. Analogously to the definition of the direct problem in Eqs. (1.a–1.c), the inverse problem can be generically defined as:

7.a $${H}_{MUT}=U\left(Indicator1, Indicator2, Indicator3\right),$$

7.b $${\varepsilon }_{\text{r}}=V\left(Indicator1, Indicator2, Indicator3\right),$$

7.c $$\text{tan}\delta =W\left(Indicator1, Indicator2, Indicator3\right).$$

Unfortunately, this time it is not possible to attain a formal definition of the functions *U*, *V* and *W*, at least with the proposed three-stage quadratic fitting method. Such a task would require closed sets of values for *Indicator1*, *Indicator2* and *Indicator3* from certain ranges with specific families of discrete possible values. Being these indicators computed from the response of the sensor, with inherently stochastic nature, such discrete values cannot be forced in practice, not even in simulations. Consequently, the inverse problem will not be defined as in Eqs. (7.a–7.c), but it has to be numerically solved from the direct problem definition in Eqs. (1.a–1.c).

**Fig. 6:**
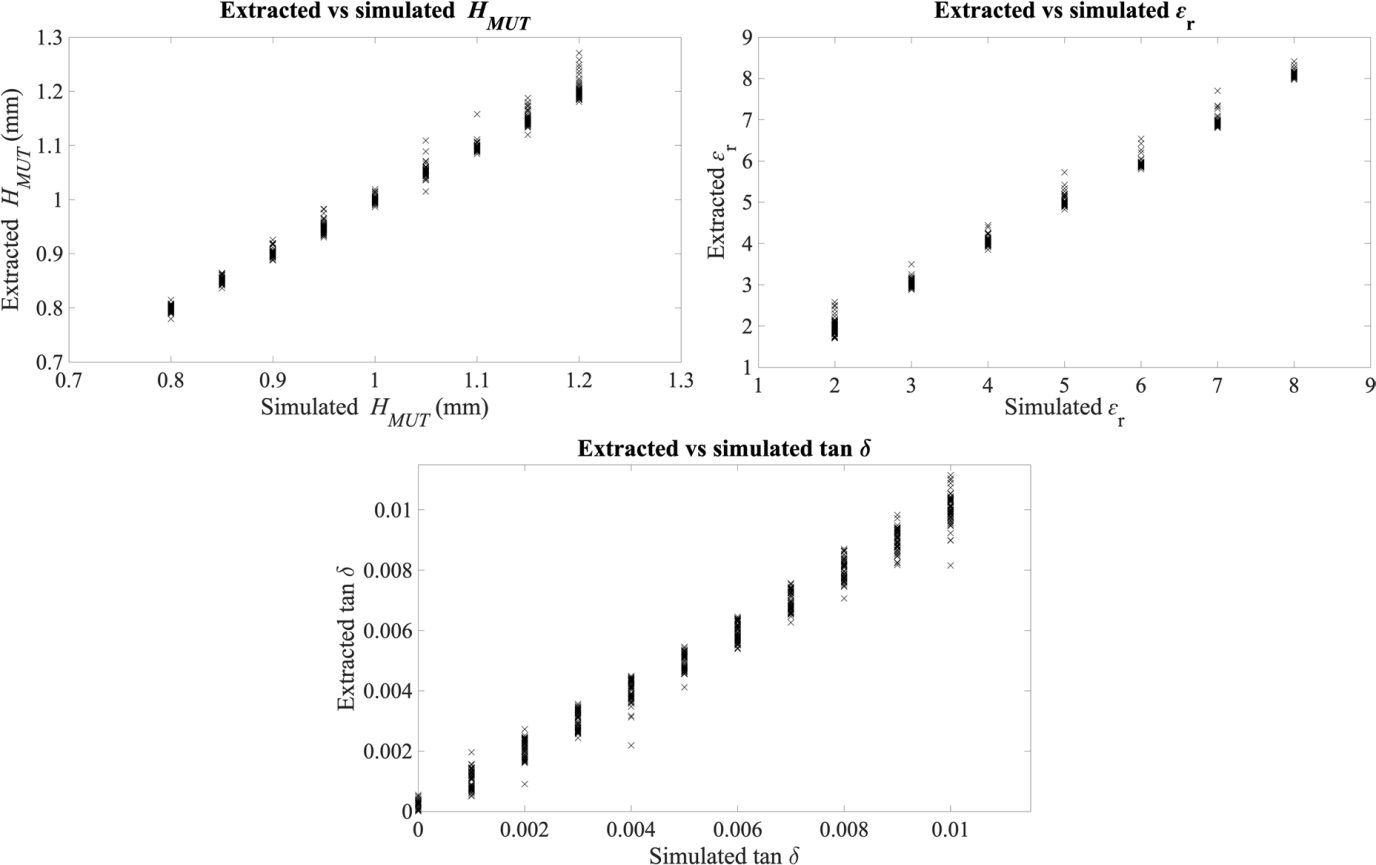
Results for the solution of the inverse problem in simulations with *set_indicators*.

As a generic solver for testing our hypothesis, MATLAB function ‘vpasolve’^49,50^ was used. This function allows to numerically solve symbolic equations for the specified variables through Newton–Raphson root-finding method^51,52^. By solving the equations in Eqs. (1.a–1.c) with the provided values of *Indicator1*, *Indicator2* and *Indicator3* and setting *H*_*MUT*_, ɛ_r_ and tan δ as unknown variables, the inverse problem can be numerically solved and the properties of the sample can be extracted. It should be noted that the solver will output all the mathematically feasible solutions, which do not need to meet the physically possible solutions. To avoid multiple solutions (from which the right one would be impossible to discern), solving ranges for each variable need to be set. In our case, bearing in mind the ranges for the properties of the samples in the simulations, the following solving ranges were set: ${H}_{MUT}\in \left[0.7, 1.3\right]$ mm, ${\varepsilon }_{\text{r}}\in \left[1.5, 11.0\right]$ and $\text{tan}\delta \in \left[0, 0.020\right]$. The results of solving the inverse problem with this procedure from the direct problem defined with *set_indicators*, having as input indicators those obtained thanks to the full-wave simulations, can be seen in Fig. 6. These plots allow us to evaluate and validate the accuracy of the proposed inverse problem solving method.

The results in Fig. 6 show a general good performance of the proposed method, being therefore feasible the detection of the properties of the sample from raw *S*-parameters with the proposed sensor and the appropriate processing. These results provisionally prove our research hypothesis, at least in simulations. Experimental validation is required for reaching sound conclusions. In the light of the results in Fig. 6, we deem the proposed solving method and the selection of indicators in *set_indicators* as validated for testing purposes. In the next section, the experimental validation of the proposed concept will be conducted.

## Results

### Implementation of the sensor

After checking the multi-parameter measurement hypothesis by comprehensive simulations, an experimental measurement campaign was carried out with assessment purposes. The proposed sensor in Fig. 2 was implemented with a microdrilling machine. For practical measurement, the interest is the extraction of the unknown properties of the sample, which are obtained by solving the inverse problem from the indicators of the response of the sensor. It should be noticed that the equations for the direct and inverse problem have been fitted thanks to data coming from simulations, and it is therefore crucial for the system to work properly that the experimental measurements resemble the simulations as much as possible. The operation frequency range selected, roughly between 1.5 and 3.0 GHz (depending on each sample), suggests that the fabrication tolerances should not have a remarkable effect. However, there are other aspects, such as the possible appearance of air bubbles or thin air layers between the samples and the substrate boards, rugosities in the samples, or misalignments between the circuit boards that could have an undesired effect in the measurement, not involved in the simulations.

**Fig. 7:**
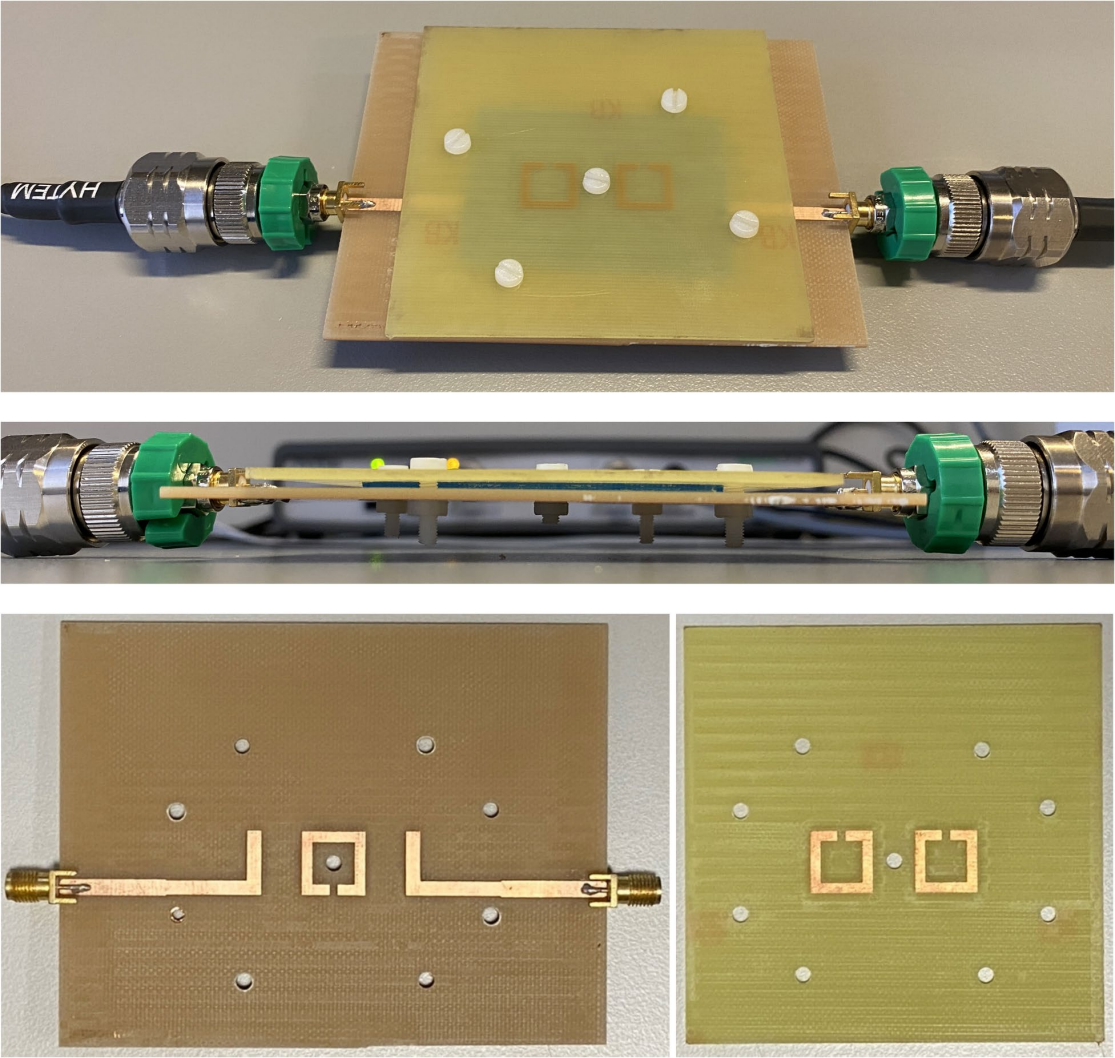
Pictures of the fabricated sensor, including the engraved sides of the substrate boards, the white nylon screws and a blue solid sample, as well as soldered SMA connectors and VNA wires.

**Fig. 8:**
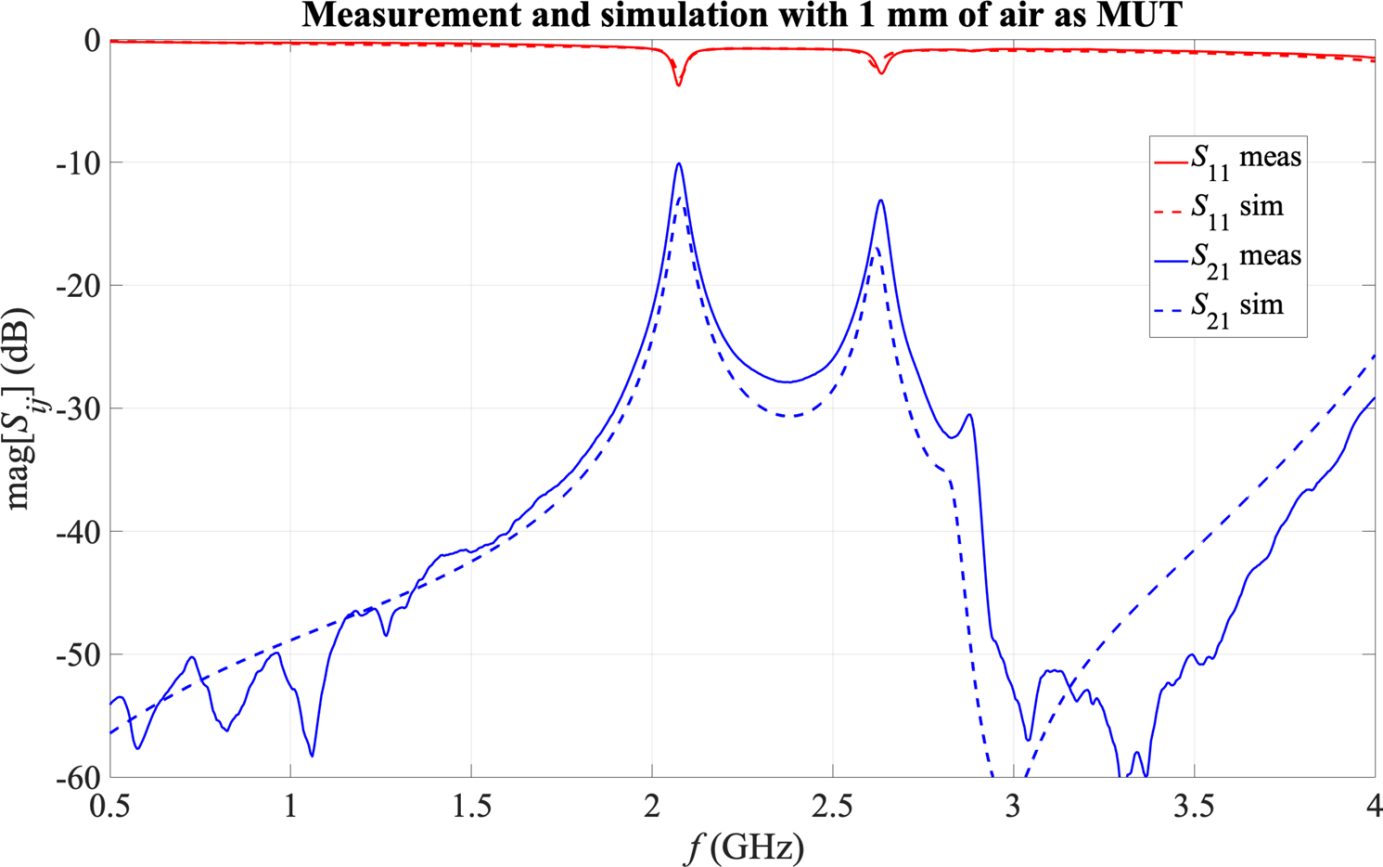
Simulated and measured response of the sensor in air-loaded state with 1 mm separation between the substrate boards.

Aiming at ensuring the right alignment of the resonators according to the model in Fig. 2, as well as preventing as much as possible the existence of air bubbles or layers and avoiding any bending of the top substrate, five 3 mm-diameter nylon screws were included in the fabricated sensor to hold and sandwich firmly the sample and align the resonators to their right positions. One screw was placed in the middle for firm holding of the structure, and the other four ones were placed next to the sample corners for good alignment of the substrate boards and the resonators and for preventing the sample from the slightest movement. Due to their strategic placement and their low-loss material (widely used in electronics), the presence of the nylon screws was checked by simulations to have no impact on the response of the sensor. A couple of pictures of the final sensor, including the screws and a solid sample, are shown in Fig. 7. SMA connectors were soldered to the I/O lines to allow for measurements with a Vector Network Analyzer (VNA). For verification, the response of the sensor in air-loaded state (i.e. without sample) and 1 mm separation between the two substrate boards (achieved thanks to 1 mm thick nylon washers in the corner screws) was measured and compared to the corresponding simulation, shown in Fig. 8, where good agreement between the measurement and the simulation is seen.

### Preparation of the samples

For experimental validation, dielectric slabs with suitable dimensions (see Table 1) were used as MUT. Two types of dielectric slabs were involved. Firstly, 8 pieces of several off-the-shelf dielectric substrates with different permittivities, losses and thicknesses were prepared, in addition to a polytetrafluoroethylene (PTFE) piece due to its interesting low-permittivity, low-loss properties. Secondly, looking for a wider study of the detection capabilities of the proposed system not limited to the properties of the commercially available substrates, 12 bespoke dielectric slabs in PolyLactic Acid (PLA) material were also prepared with tailored *H*_*MUT*_, ɛ_r_ and tan δ values by means of the 3-D printing technique described in Ref.^53^, using different filling densities to achieve different dielectric properties. Before any measurement, all these samples were carefully polished to reduce as much as possible their rugosity (it is worthwhile mentioning that a hard polishing could not be applied due to breakage risk, especially for the brittle PLA samples). Also, a hole of 3.2 mm diameter was drilled into the centre of each sample for the nylon screw. For trustable verification of the results, the *H*_*MUT*_ of each sample was accurately measured (after polishing) by a micrometre (ref. 3358-25 from INSIZE Europe, Derio, Spain), and the ɛ_r_ and tan δ values for each sample were empirically measured with the measurement cell shown in Ref.^3^, verifying a general good agreement with the datasheets of the commercial substrates and with the predictions of the method shown in Ref.^53^ for the PLA samples. Table 2 offers a summary of the 21 samples involved as MUT in this study and their properties (the uncertainties are ± 0.01 mm for *H*_*MUT*_, ± 0.05 for ɛ_r_ and ± 0.005 for tan δ). From them, three representative ones were taken as calibration samples, as described in section “Methods”.

**Table 2:** Properties of the 21 samples involved in the experimental validation.

Sample no.	*H*_*MUT*_ (mm)	ɛ_r_	tan δ	Material	Calibration
1	0.96	2.46	0.016	3-D-printed PLA	
2	1.30	2.32	0.015	3-D-printed PLA	
3	1.12	2.31	0.018	3-D-printed PLA	
4	1.67	2.16	0.015	3-D-printed PLA	X
5	0.97	2.53	0.018	3-D-printed PLA	
6	1.11	2.62	0.018	3-D-printed PLA	
7	1.30	2.45	0.018	3-D-printed PLA	
8	1.60	2.39	0.018	3-D-printed PLA	
9	1.29	3.00	0.020	3-D-printed PLA	
10	1.07	3.00	0.020	3-D-printed PLA	
11	0.86	3.05	0.020	3-D-printed PLA	
12	1.56	2.75	0.020	3-D-printed PLA	
13	0.41	4.58	0.021	FR4 substrate^54^	
14	0.75	4.78	0.021	FR4 substrate^54^	X
15	1.53	4.66	0.021	FR4 substrate^54^	
16	0.99	3.06	0.001	Rogers AD255C substrate^55^	
17	0.61	9.24	0.004	Taconic RF-10 substrate^56^	X
18	0.80	5.59	0.004	Taconic RF-60TC substrate^57^	
19	1.19	5.76	0.003	Taconic RF-60TC substrate^57^	
20	1.59	2.32	0.000	PTFE^58^	
21	0.77	2.77	0.002	Taconic TLX-8 substrate^59^	

### Measurements and results

All the experimental samples in Table 2 were measured with the fabricated sensor. A proper calibration was applied to each experimental measurement, as described in section “Methods”. After this procedure, the 18 experimental measurements could be processed and analysed by solving the inverse problem from the direct problem defined by *set_indicators*, as discussed earlier. Again, solving ranges needed to be defined for each variable (*H*_*MUT*_, ɛ_r_ and tan δ) so that the solver would output a unique solution for each measurement. Considering that the experimental samples had larger values for the three variables than the simulations (due to substrates availability and 3-D-printing possibilities), the largest ranges to ever output single solutions for the 18 measurements were explored. The resulting ranges were ${H}_{MUT}\in \left[0.1, 1.7\right]$ mm, ${\varepsilon }_{\text{r}}\in \left[1.5, 10.0\right]$ and $\text{tan}\delta \in \left[-0.003, 0.035\right]$, thus leading to acceptable ranges for a wide variety of scenarios and applications requiring the characterization of solid slabs. It should be noted that, in order to obtain a solution for the samples with very low values of tan δ, a slight range of negative values had to be allowed, due to the usual fluctuations of the root-finding algorithms in addition to the truncation and digital errors. Of course, these negative values are not physically possible, but they are mathematically required for the solutions to converge. Due to this reason, the solution value for tan δ is always down-limited to zero.

**Fig. 9:**
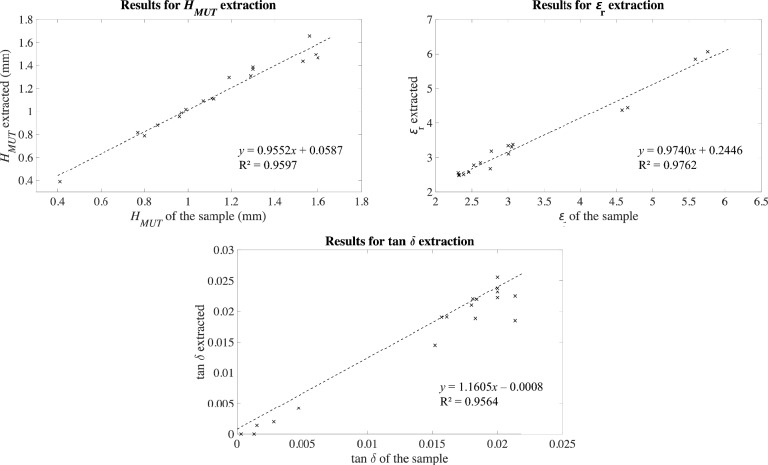
Experimental results for the multi-parameter detection of the properties of the sample, along with the resulting fitting lines.

The simultaneously extracted properties of the samples in the 18 measurements resulting from the solution of the inverse problem with these solving ranges are plotted in Fig. 9 against the actual properties of the sample. The experimental mean errors for each property of the sample were ± 0.05 mm for *H*_*MUT*_, ± 0.215 for ɛ_r_, and ± 0.0022 for tan δ.

## Discussion

The results in Fig. 9 show good sensing capabilities as for the simultaneous extraction of the three targeted properties from the sample with the proposed sensor, including acceptable errors and wide measurement ranges. This result allows us to experimentally validate the main hypothesis of this study. The idea that three different parameters can be extracted from the sample with a sensor involving three coupled resonators is reasonable, and the proposed analytical method for the simultaneous extraction of these parameters from one measurement has been proved effective with simulations (Fig. 6) and measurements (Fig. 9).

At this point, it is interesting to discuss some limitations of this study for a moderated interpretation of the results. It should be mentioned that, for building the direct problem from which the characteristics of the sample are extracted with an inverse solver, a given order was arbitrarily chosen for the proposed three-stage quadratic curve fitting procedure (first take a fixed value of tan δ, then fit curves against *H*_*MUT*_ for each value of ɛ_r_, then fit new curves for the previously obtained coefficients against the different values of tan δ, see Fig. 4), but a different one could have been chosen. This procedure was selected only for hypothesis validation, with no aim of optimization of the measurement capabilities. Second-order polynomials for the fitting curves were chosen under the same rationale, also looking for acceptable computation times. In this sense, the painstaking study of the optimal order of the polynomials, the optimal fitting functions, or the optimal order for the multi-stage fitting process, among any other influencing factors, raises as highly desirable now that the sensing concept has been validated.

Efforts on the optimization of other interesting aspects affecting the final measurement capabilities, such as optimal selection, maybe through appropriate metrics from information theory, of the sensor response indicators to be considered in Eqs. (1.a–1.c) (*set_indicators* = (*Q*_u_^p1^, *f*_r_^p3^, ∆*f*) in our case) could also be worthwhile, as well as research on more convenient inverse solving methods, both from the accuracy and the computational load points of view. The study of more sophisticated topologies for the sensor implementation, leveraging the impact of the MUT in the electromagnetic response and optimizing this impact with respect to the selected indicators, direct/inverse problem functions or solving method also raises as appealing but challenging. These aspects fell out of the scope of this exploratory work, and were left as potential future lines.

## Methods

For each experimental measurement, once the sensor was loaded with the corresponded sample, the response of the sensor in terms of the scattering parameters was recorded with a properly calibrated VNA (Anritsu ref. MS46122A, Atsugi-shi, Japan). Despite the good agreement between measurement and simulation for the sensor in air-loaded state (see Fig. 8), a certain frequency shift was seen for all the measurements involving a solid MUT. Considering the transmission parameter *S*_21_, as shown in Fig. 3, very similar signal shapes were observed for measurements and simulations, as well as very similar signal amplitude levels, but there was always a frequency shift. This shift was not constant, but it seemed to be linked to the effective permittivity of the sample, which is logical considering the relationship between the resonance frequency of a microwave resonator and the effective permittivity of the medium^60^.

Indeed, the impact of the possible error in the estimation of the *H*_*MUT*_ of the sample with the micrometre, which could affect the shape of the final response of the sensor (by means of variations of the couplings), can be considered as negligible. Also, variations in the losses could be seen as variations in the signal levels, which did not appear. This effect of frequency shift can be consequently attributed to an effective permittivity of the sample different from the expected ɛ_r_ according to Table 2. This effect is understandable considering the sandwich structure and the likelihood of having thin, irregular air layers between the sample and the two substrate boards. The fact that a hard polishing could not be applied to the samples (due to their inherent mechanical properties) and the non-neglectable thickness of the copper lines etched in the substrate also supports this idea. Fortunately, this effect can be characterized and compensated.

**Fig. 10:**
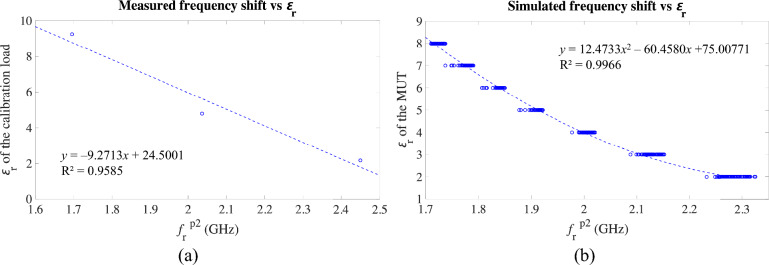
Plots of the ɛ_r_ of the sample against *f*_r_^p2^ for calibration purposes. (**a**) Experimental measurement of the three calibration loads. (**b**) Simulations (the multiple points are due to the discrete values of ɛ_r_ associated to different values of *H*_*MUT*_ and, less noticeably, of tan δ).

To do this, as it is common for most pieces of electronic measurement equipment, a calibration procedure for the practical measurements is required, especially for a system like this one using data extraction functions fitted with ideal simulation data. In our case, three samples from the set in Table 2 were selected as calibration samples, henceforward referred to as calibration loads, thereby leaving the remaining 18 samples for experimental validation. Namely, samples #4, #14 and #17 were selected as calibration loads, and they were considered as ideally known. It should be noted that these calibration loads, with ɛ_r_ values of 2.164, 4.784 and 9.237, were selected in order to fully cover the target permittivity range, and they also reflect the diversity of the samples as for the loss tangent and the *H*_*MUT*_ (see Table 2). This selection of calibration loads is also useful for having a non-linear approximation in order to make the simulations fit the measurements. Deepening in the idea that the shift to be corrected is due to effective permittivity mismatch, and considering the close relationship between effective permittivity and resonance frequency, the relationship between the known ɛ_r_ of the three calibration samples and the associated measured centre resonance frequency (*f*_r_^p2^) was analysed. As shown in Fig. 10a, a direct linear relationship could be established with a confidence of 95.85%. Since the interest is in the measurements to approach the simulations, this dependence of the simulated *f*_r_^p2^ on the ɛ_r_ of the sample could also be plotted, which can be described by a second order polynomial with a confidence of 99.66%, as shown in Fig. 10b.

In fact, for calibration purposes, what we are interested in is a relationship that allows us to obtain the frequency shift, i.e. *f*_r_^p2^_measured_ − *f*_r_^p2^_simulated_, in terms of the ɛ_r_ of the sample. This can be done by means of the inverse relationships to those in Fig. 10 (i.e. *f*_r_^p2^ vs ɛ_r_). These inverse relationships were found to be:

8.a $${{{f}_{\text{r}}}^{\text{p}2}}_{\text{measured}}=-0.1034{\varepsilon }_{\text{r}}+2.6184, {R}^{2}=0.9585,$$

8.b $${{{f}_{\text{r}}}^{\text{p}2}}_{\text{simulated}}=0.0101{{\varepsilon }_{\text{r}}}^{2}-0.1918{\varepsilon }_{\text{r}}+2.6208, {R}^{2}=0.9956.$$

**Fig. 11:**
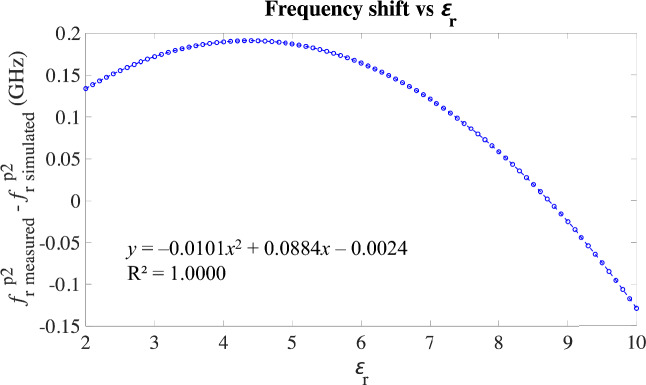
Experimental frequency shift depending on the ɛ_r_ of the sample.

The equations in (8.a, 8.b) allow to easily compute the expected value of *f*_r_^p2^ for a large number of values of ɛ_r_ both in simulations and measurements, without any need to simulate or measure. Then, for each value of ɛ_r_ the pair of values obtained for *f*_r_^p2^_measured_ and *f*_r_^p2^_simulated_ can be subtracted, thereby obtaining the expected frequency shift for a large set of values of ɛ_r_. This was done for ɛ_r_ ranging 2–10 in steps of 0.1, resulting in the relationship in Fig. 11.

The relationship in Fig. 11 allows to define the frequency shift (and thereby to correct it) according to the ɛ_r_ of the sample, regardless the effective permittivity in the system. Let $\overrightarrow{f}$ be the frequency vector of any experimental measurement, then according to Fig. 11:

9 $${\overrightarrow{f}}_{\text{corrected}}={\overrightarrow{f}}_{\text{measured}}+0.0101{{\varepsilon }_{\text{r}}}^{2}-0.0884{\varepsilon }_{\text{r}}+0.0024.$$

**Fig. 12:**
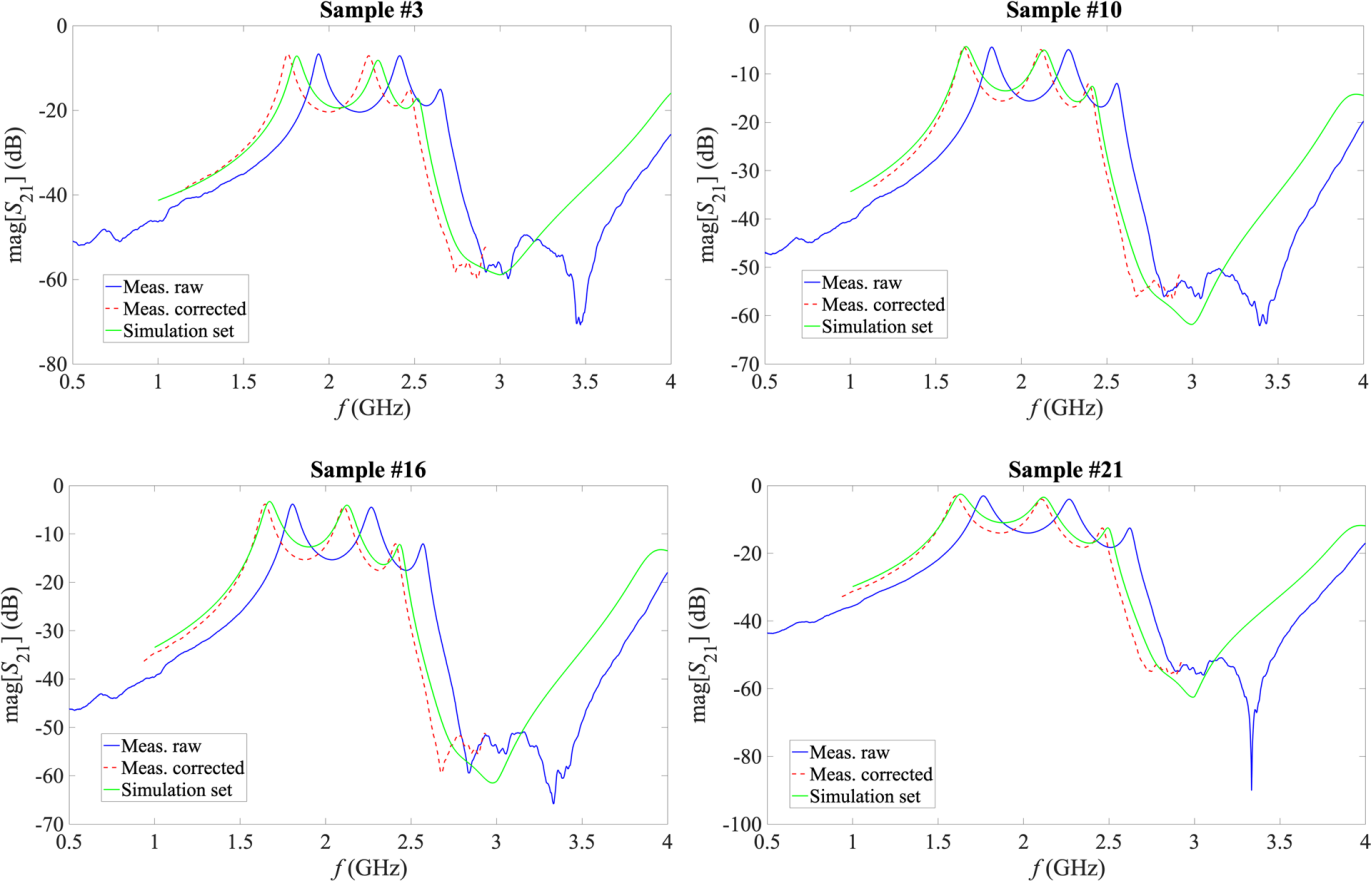
Examples of some measurements, including the raw measurement, the corrected measurement and the closest simulation.

However, the correction in Eq. (9) depends on an unknown parameter, the ɛ_r_ of the sample. For effective calibration of the measurement, this dependence needs to be removed thanks to the calibration relationship obtained from the calibration loads, as shown in Fig. 10a. Applying this relationship to Eq. (9) yields:

10 $${\overrightarrow{f}}_{\text{corrected}}={\overrightarrow{f}}_{\text{measured}}+0.8682{{{{f}_{\text{r}}}^{\text{p}2}}_{\text{bef}}}^{2}-3.7688{{{f}_{\text{r}}}^{\text{p}2}}_{\text{bef}}+3.8992,$$

where ${{{f}_{\text{r}}}^{\text{p}2}}_{\text{bef}}$ is the measured resonance frequency of the second peak before any correction to the frequency vector, ${\overrightarrow{f}}_{\text{measured}}$ is the raw frequency vector of the measurement, and ${\overrightarrow{f}}_{\text{corrected}}$ is the finally corrected frequency vector. The correction in Eq. (10) effectively allows to correct the frequency shift due to practical effective permittivity mismatch in the experimental measurements by only using parameters from the raw measurement. As an example, Fig. 12 plots some raw measurements together with the corrected measurement according to Eq. (10) and with the response obtained in the simulation of the sample with the closest parameters to the experimental one from the simulations set.

## Data Availability

Data is provided within the manuscript and it can be downloaded at https://medicalrobotics.umh.es/datarepository/.
